# L-carbocisteine can cause cholestasis with vanishing bile duct syndrome in children: A case report

**DOI:** 10.1097/MD.0000000000031486

**Published:** 2022-11-11

**Authors:** Yugo Takaki, Makoto Murahashi, Kei Honda, Katsuki Hirai

**Affiliations:** a Department of Pediatrics, Japanese Red Cross Kumamoto Hospital, Kumamoto, Japan.

**Keywords:** children, cholestasis, drug-induced lymphocyte stimulation tests, L-carbocisteine, vanishing bile duct syndrome

## Abstract

Vanishing bile duct syndrome (VBDS) is the acquired progressive destruction and disappearance of intrahepatic interlobular bile ducts in the absence of underlying liver or biliary tract disease, causing chronic cholestasis. Infections, drugs, toxins, malignant diseases, and certain immunological processes are associated with the development of this syndrome. There have been no reports of children developing VBDS as a consequence of the administration of L-carbocisteine.

**Patient concerns:**

A 9-year-old Japanese girl presented with fever, jaundice, and skin rash. Laboratory investigations revealed elevated levels of serum transaminases, γ-glutamyltransferase, and bilirubin. Histopathological features were consistent with a diagnosis of VBDS. Drug-induced lymphocyte stimulation tests (DLST) were positive for L-carbocisteine.

**Diagnosis:**

VBDS caused by L-carbocisteine.

**Interventions:**

Ursodeoxycholic acid and discontinuation of L-carbocisteine.

**Outcomes:**

The patient responded to treatment based upon discontinuation of L-carbocisteine and administration of ursodeoxycholic acid. Her transaminase and bilirubin levels were normalized gradually.

**Lessons:**

Physicians should be aware of the fact that L-carbocisteine can cause cholestasis with VBDS in children.

## 1. Introduction

L-carbocisteine is a mucolytic agent used by many pediatricians as medication for common cold. These agents have various physiological functions, including antioxidant and anti-inflammatory effects and modulation of mucin expression induced by lipopolysaccharide.^[1]^ L-carbocisteine can cause severe liver damage in adults; however, there have been few reports of serious liver damage in children.

Vanishing bile duct syndrome (VBDS) is characterized by progressive destruction and disappearance of intrahepatic interlobular bile ducts in the absence of an underlying liver or biliary tract disease, causing chronic cholestasis. Infections, drugs, toxins, and oncologic and immunological processes are associated with exposure.^[2]^ Over 30 drugs have been reported to cause VBDS in adults, and several cases of drug-induced VBDS have been reported in children.^[3]^

Here, we report the case of a healthy, 9-year-old Japanese girl who presented with cholestasis and VBDS caused by L-carbocisteine.

## 2. Case presentation

A healthy 9-year-old Japanese girl was referred to our hospital with a chief complaint of persistent fever. She was born following an uncomplicated pregnancy and delivery, to healthy parents without consanguinity. Three days before admission, she had a mild fever and was treated with oral acetaminophen (320 mg/day, 10.7 mg/kg), cefcapene (300 mg/day, 10 mg/kg), and L-carbocisteine (1500 mg/day, 50 mg/kg) for 3 days, under a presumptive diagnosis of upper respiratory tract infection, at a private clinic. Her medical history was not significant. There were no other medication exposures in the months prior to the onset of symptoms. Three days after the onset of fever, the patient was referred to our hospital.

On admission, she was febrile and had a skin rash. Physical examination revealed a body temperature of 38.0°C, heart rate of 124 beats/min, respiratory rate of 34/min, and blood pressure of 104/61 mm Hg. Upon admission, all previous medications, including L-carbocisteine, were discontinued.

Her initial laboratory results included serum aspartate aminotransferase, 238 U/L (normal range, <30 U/L); alanine aminotransferase, 237 U/L (<23 U/L); γ-glutamyl transferase, 269 U/L (<32 U/L); total/direct bilirubin (TB/DB), 2.5/2.08 mg/dL (<1.5/<0.3 mg/dL); albumin, 3.9 g/dL (4.1–5.1 g/dL); total bile acids, 130.8 μmol/L (<10 μmol/L); fasting plasma glucose, 90 mg/dL (<109 mg/dL), and prothrombin time-international normalized ratio, 1.18 (0.85–1.15). The complete blood cell count was normal. The infectious disease workup for hepatitis A IgM, hepatitis B surface antigen, hepatitis C RNA polymerase chain reaction, Epstein Barr virus IgM, cytomegalovirus IgM, herpes simplex virus IgM, mycoplasma particle agglutination, and parvovirus IgM did not reveal any causative infectious agent. The ceruloplasmin level was normal (49 mg/dL). Abdominal ultrasonography showed a normal liver span for her age, with a normal common biliary duct and a thickened gallbladder wall. At 3 days after admission, she developed an erythematous macular rash, chapped lips, and respiratory distress; she received intravenous immunoglobulin for presumed Kawasaki disease. Her respiratory status necessitated oxygen support. Chest radiography and computed tomography showed consolidation and ground-glass opacity in both lungs. At 5 days after admission, she developed genital ulcers, icteric sclera, and progressive jaundice. Therefore, we suspected Stevens–Johnson syndrome and administered prednisolone (30 mg/day, 1 mg/kg). Treatment with ursodeoxycholic acid (UDCA; 200 mg/day, 6.6 mg/kg) was initiated on day 6 of hospitalization.

**Figure 1.**
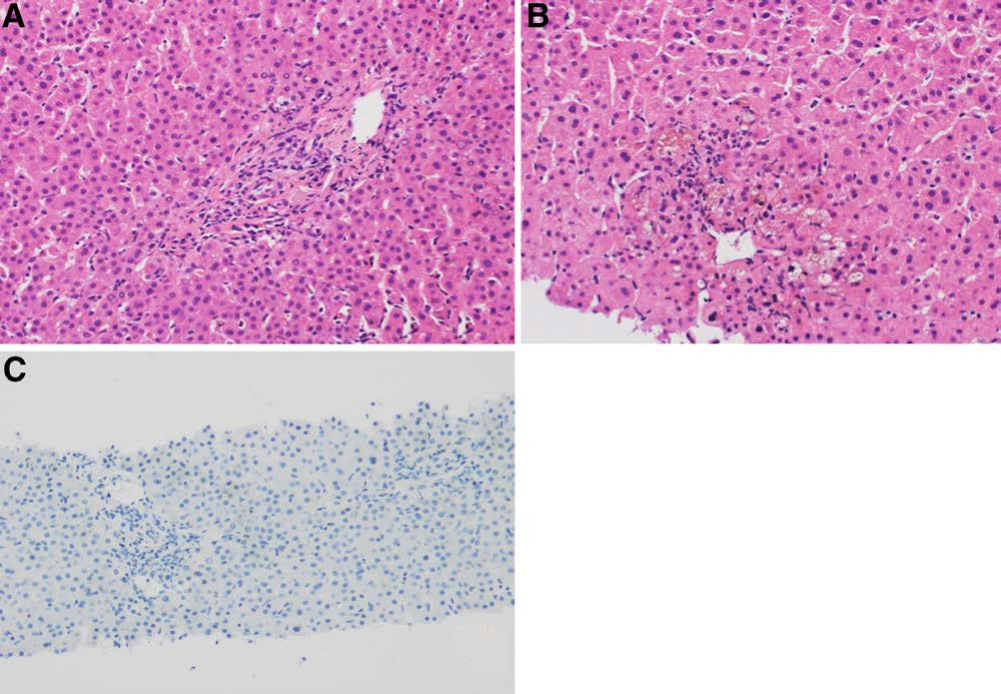
**Histologic findings.** (A) Hematoxylin and eosin stain (H&E, ×200). Liver histology results show the portal tract with lack of interlobular bile ducts. (B) Hematoxylin and eosin stain (H&E, ×200). Bile plugs around the central veins. (C) Cytokeratin 19 stain (CK19, ×100). Immunostaining for cytokeratin 19 shows loss of normal interlobular bile duct around the portal tracts.

The patient’s fever improved, and her respiratory status improved gradually; however, liver biopsy was performed 22 days after the onset of jaundice owing to continued presence of features of cholestasis. Laboratory results revealed serum TB/DB of 16.9/12.98 mg/dL and γ-glutamyltransferase levels of 442 IU/L. Histolopathological examination of the biopsy specimen revealed biliary stasis around the central vein, with disappearance of the interlobular bile ducts. Cytokeratins were not observed on immunohistochemical evaluation. Macro steatosis was not observed in the biopsy tissue, but focal cholestasis was seen within the hepatocytes. There was no portal tract or lobular inflammation. Periodic acid-Schiff staining showed normal glycogen. These findings were consistent with those of VBDS (Fig. 1).

**Figure 2.**
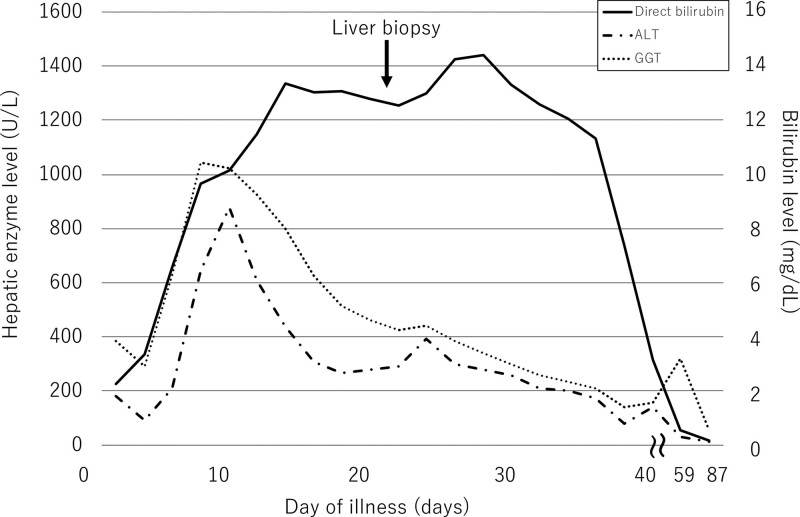
**Direct bilirubin, γ-glutamyl transferase, and alanine aminotransferase levels on each day of illness.** ALT = alanine aminotransferase, GGT = γ-glutamyl transferase.

After the diagnosis of VBDS was confirmed, jaundice and fatigue persisted for more than 1 week. The total bilirubin levels peaked at 17.8 mg/dL, with direct bilirubin levels of 14.27 mg/dL. Subsequently, there was an improvement in cholestasis (Fig. 2).

After medication was stopped, we performed drug-induced lymphocyte stimulation tests (DLSTs) twice each for L-carbocisteine, acetaminophen, and cefcapene. The second DLST was positive for L-carbocisteine (192%; 8 months after discontinuation of L-carbocisteine), whereas the DLSTs for all other medicines were negative. L-carbocisteine was not administered again, and her hepatic test results remained normal at the 16 month follow-up examination.

## 3. Discussion

We present the case of a 9-year-old girl with VBDS caused by the administration of L-carbocisteine. The discontinuation of L-carbocisteine, with UDCA treatment, were successful in improving the patient’s condition.

Drug-induced liver injury (DILI) is an idiosyncratic cause of both acute and chronic liver diseases. These pathologies can initially present asymptomatically and are characterized by hepatocellular, cholestatic, or mixed phenotypes.^[2]^ VBDS is defined as a reduced ratio of the interlobular ducts to portal tracts, usually <0.5. Alagille syndrome, viral infections, cystic fibrosis, progressive familial intrahepatic cholestasis, primary sclerosing cholangitis, and familial idiopathic adult ductopenia can cause bile duct paucity. The typical presentations include jaundice, abdominal pain, pruritus, weight loss, cholestasis, and elevated transaminase levels.

VBDS is a histopathological diagnosis and is found on evaluation of patients with DILI.^[4]^ This acquired condition is associated with more than 30 medications, including amoxicillin, nonsteroidal anti-inflammatory drugs, steroids, erythromycin, and tricyclic antidepressants. To the best of our knowledge, L-carbocisteine has not been reported to be associated with VBDS to date. In children, a few cases of VBDS induced by amoxycillin-clavulanic acid, ibuprofen, valproic acid, carbamazepine, lamotrigine, trimethoprim-sulfamethoxazole, and pembrolizumab have been reported.^[3,5–13]^ Our patient presented with jaundice, pruritus, weight loss, cholestasis, and elevated transaminase levels; however, we could not identify these symptoms as DILI at the beginning. The primary reason for this was the short duration of medication (only 3 days). The second reason is that these medicines (i.e., L-carbocisteine, acetaminophen, and cefcapene) are not generally known to cause DILI, especially cholestasis or VBDS. We established the fact that VBDS was the cause of cholestasis by histopathological evaluation of the liver biopsy specimen obtained from the patient.

DILI occurs in susceptible individuals through a combination of genetic and environmental risk factors that may modify drug metabolism and/or excretion, thus leading to a cascade of cellular events, including oxidative stress, apotosis/necrosis, haptenization, immune response activation, and a failure to adapt. Fontana described that intrinsic and idiosyncratic DILI cases are commonly thought to arise by different pathophysiologic mechanisms.^[14]^ Intrinsic hepatotoxins, such as acetaminophen, are typically dose dependent. In contrast, most instances of DILI observed in clinical practice are termed idiosyncratic and are not clearly related to the dose, route, or duration of drug administration.^[14,15]^ In our case, the duration from the initiation of L-carbocisteine administration to the onset of the symptom was unusually short. Therefore, the mechanism of our case was presumed as idiosyncratic type.

The treatment for VBDS involves discontinuation of the causative medication and prevention of re-exposure, but it can take months for laboratory values to normalize.^[2]^ The symptomatic treatment of cholestasis and pruritus includes administration of cholestyramine, UDCA, rifampicin, and opiate antagonists. Corticosteroids have not been proven to be effective.^[5]^ There is evidence that UDCA exerts its therapeutic effect through multiple mechanisms, including choleresis, the protection of hepatocellular lipid membranes against hydrophobic cytotoxic bile acids, and the suppression of bile acid-induced apoptosis of the liver. Ursodeoxycholic acid also improves canalicular transport by restoring bile flow, promotes excretion of toxic hydrophobic bile salts from hepatocytes, and prevents hepatocyte necrosis and apoptosis.^[16]^ In our case, discontinuation of L-carbocisteine and administration of ursodeoxycholic acid resulted in improvement in the patient’s condition; however, she took several months to reach normalcy. VBDS can progress despite discontinuation of the offending medications. However, the transaminase and bilirubin levels can show a return to normal levels despite persistent loss of the bile ducts.^[17]^

Some studies have reported that severe DILI-like acute liver failure requires liver transplantation.^[7,12,17]^ In severe cases, therapies for VBDS have been attempted with the aim of blunting the immune response. Tacrolimus, intravenous immunoglobulin, tumor necrosis factor-α blockade, and plasmapheresis have been used in adults with VBDS. White et al used steroids, intravenous immunoglobulin, plasmapheresis, and infliximab in a 6-year-old patient with VBDS secondary to toxic epidermal necrolysis; unfortunately, the patient did not survive.^[17]^ It is also difficult to predict the severity of VBDS.

L-carbocisteine has been widely and safely used in Japan since 1981 in patients with mucus sputum due to acute bronchitis or chronic obstructive lung disease. This includes adults as well as children. We could not find any previous case report of L-carbocisteine-induced liver injury. Kudo et al reported a definitive case of L-carbocisteine-induced pneumonia, which was diagnosed using DLSTs of L-carbocisteine.^[18]^ They also mentioned that this patient had L-carbocisteine-induced pneumonia twice within 3 years. After the second episode, she was diagnosed with L-carbocisteine-induced pneumonia.

In the present case, DLST worked decisively for the definitive diagnosis of DILI. DLST is an in vitro immunological examination that inspects the proliferation of T cell in response to a suspected drug. It is safe, and its results can be used in comparison with those of classical in vivo sensitization tests; therefore, it has already been considered as a reliable diagnostic modality for DILI.^[19]^ The main advantage of this test is its applicability with many different drugs in different immune reactions. The main disadvantage is that its sensitivity is limited, despite being higher than that of other tests for drug hypersensitivity diagnosis. Aiso et al performed a prospective study of 307 DILI cases between 2010 and 2018 in Japan. Interestingly, DLST was performed in 59% of cases and was positive in 48% and semipositive in 3% of cases.^[20]^ Although some concern regarding its technical sensitivity and specificity still exists, DLST can be helpful for diagnostic purposes.^[21]^

To the best of our knowledge, this is the first report of a cholestatic child with VBDS caused by L-carbocisteine and the first case responsive to UDCA therapy. Severe DILI, including VBDS, should be considered in patients taking L-carbocisteine who have elevated serum transaminase or bilirubin levels and should prompt referral to a pediatric gastroenterologist.

## 4. Conclusion

We report the case of a school-going girl with cholestasis and VBDS caused by L-carbocisteine. It is important for physicians to be aware that L-carbocisteine can cause cholestasis in VBDS. Features of hepatotoxicity developing in a patient consequent to the administration of L-carbocisteine should prompt a referral to a pediatric gastroenterologist for evaluation and further management.

## Author contributions

YT contributed to the concept and design of the study. YT and MM analyzed and interpreted the data. YT wrote the manuscript, while MM, KH1, and KH2 edited it. YT and KH2 supervised the study and reviewed the manuscript. Thus, all authors contributed to the study.

**Conceptualization:** Yugo Takaki.

**Data curation:** Yugo Takaki, Makoto Murahashi, Kei Honda, Katsuki Hirai.

**Formal analysis:** Yugo Takaki.

**Investigation:** Yugo Takaki, Makoto Murahashi, Kei Honda, Katsuki Hirai.

**Methodology:** Yugo Takaki.

**Project administration:** Katsuki Hirai.

**Supervision:** Makoto Murahashi, Kei Honda, Katsuki Hirai.

**Validation:** Yugo Takaki.

**Writing – original draft:** Yugo Takaki.

**Writing – review & editing:** Makoto Murahashi, Kei Honda, Katsuki Hirai.

## Acknowledgments

We thank the patient and her parents for their cooperation, as well as Dr Michiko Nagamine and Dr Tsuguharu Asato at Japanese Red Cross Kumamoto Hospital (for pathological diagnosis advice), Dr Hironori Kusano at Kurume University School of Medicine (for pathological diagnosis advice), and Tatsuki Mizuochi at Kurume University School of Medicine (for clinical advice).
